# Calcium Homeostasis in the Epididymal Microenvironment: *Is Extracellular* C*alcium a Cofactor for Matrix Gla Protein-Dependent Scavenging Regulated by Vitamins*

**DOI:** 10.3389/fcell.2022.827940

**Published:** 2022-02-17

**Authors:** Winnie Shum, Bao Li Zhang, Albert Shang Cao, Xin Zhou, Su Meng Shi, Ze Yang Zhang, Lou Yi Gu, Shuo Shi

**Affiliations:** ^1^ School of Life Science and Technology, ShanghaiTech University, Shanghai, China; ^2^ Center for Excellence in Molecular Cell Science, Shanghai Institute of Biochemistry and Cell Biology, Chinese Academy of Sciences, Shanghai, China; ^3^ NHC Key Lab of Reproduction Regulation, Shanghai Institute for Biomedical and Pharmaceutical Technologies, Reproduction and Development Institution, Fudan University, Shanghai, China; ^4^ Shanghai Institute for Advanced Immunochemical Studies, ShanghaiTech University, Shanghai, China

**Keywords:** sperm maturation, epididymis, calcium homeostasis, luminal microenvironment, GGCX, matrix gla protein (MGP), Trpv6, Tmem16a

## Abstract

In the male reproductive tract, the epididymis is an essential organ for sperm maturation, in which sperm cells acquire mobility and the ability to fertilize oocytes while being stored in a protective microenvironment. Epididymal function involves a specialized luminal microenvironment established by the epithelial cells of epididymal mucosa. Low-calcium concentration is a unique feature of this epididymal luminal microenvironment, its relevance and regulation are, however, incompletely understood. In the rat epididymis, the vitamin D-related calcium-dependent TRPV6-TMEM16A channel-coupler has been shown to be involved in fluid transport, and, in a spatially complementary manner, vitamin K2-related γ-glutamyl carboxylase (GGCX)-dependent carboxylation of matrix Gla protein (MGP) plays an essential role in promoting calcium-dependent protein aggregation. An SNP in the human *GGCX* gene has been associated with asthenozoospermia. In addition, bioinformatic analysis also suggests the involvement of a vitamin B6-axis in calcium-dependent MGP-mediated protein aggregation. These findings suggest that vitamins interact with calcium homeostasis in the epididymis to ensure proper sperm maturation and male fertility. This review article discusses the regulation mechanisms of calcium homeostasis in the epididymis, and the potential role of vitamin interactions on epididymal calcium homeostasis, especially the role of matrix calcium in the epididymal lumen as a cofactor for the carboxylated MGP-mediated scavenging function.

## General Introduction

**FIGURE 1:**
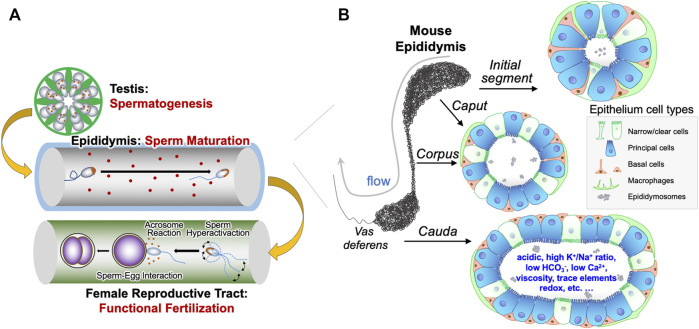
Epididymal epithelial cells and luminal microenvironment are important for male reproduction. **(A)** Graphic illustration of the generation of spermatozoa in the testis through spermatogenesis, the maturation of spermatozoa in the epididymis, and the spermatozoa functions, including sperm hyperactivation, acrosome reaction and sperm-egg interaction, in the female reproductive tract required for fertilization. **(B)** The different cell types in the various regions of epididymis, including basal cells, clear cells, narrow cells, and principal cells, as well as immunological cells from systemic circulation. These different cell types work in a concerted manner to form a unique luminal microenvironment that promotes maturation of sperm during its epididymal transit and ensures male fertility.

The production of viable and functionally competent spermatozoa is a prerequisite for male fertility. This is achieved through normal spermatogenesis in the testis and maturation of spermatozoa in the epididymis. Spermatozoa are non-fertilising when released from the testis but become functionally competent during epididymal transit. The fertilizing competence of spermatozoa is conferred by their interaction with the epididymal luminal microenvironment, which is formed by the epithelial cellular activities and retained behind the blood-epididymis barrier (Dacheux and Dacheux, 2014; Dube and Cyr, 2012; Robaire and Hinton, 2015; Zhou et al., 2018). Spermatozoa therefore mature and are protected in a special physiologically and immunologically privileged epididymal microenvironment, in which they undergo a series of tightly-controlled sequential maturational processes in the precisely segmented luminal compartments (Cornwall, 2009; Gervasi and Visconti, 2017; Gregory and Cyr, 2014; Hermo, 2002; Mital et al., 2011; Pleuger et al., 2020; Robaire and Hinton, 2015; Voisin et al., 2019; Wong P. Y. D. et al., 2002; Zhou et al., 2018). Through these maturation processes, spermatozoa acquire the motility to swim within the female tract and to undergo a three-stage modification process that enables oocyte fertilization (Aitken, 2016; Breitbart, 2002; Reid et al., 2011; Stival et al., 2016), including capacitation and hyperactivation (Aitken and Nixon, 2013; Gervasi and Visconti, 2016), as well as the acrosome reaction (Brucker and Lipford, 1995) (Figure 1A). The minimal time for spermatozoa to transit through the entire epididymis usually takes 1–16 days, depending on the species. In humans, the average transit time is approximately 1–2 days (Amann and Howards, 1980), and spermatozoa can be stored in the cauda epididymidis for several days and even months (Robaire and Hinton, 2015). Although fertilization occurs within the female genital tract, the functional ability for fertilization is acquired by spermatozoa when they transit through the highly convoluted epididymal tubule, where spermatozoa mature but remain in a dormant stage (Figure 1B). Hence, the epididymis plays a vital role for sperm maturation and male reproduction.

Epididymal function relies on a highly specialized epididymal luminal microenvironment, which is formed and maintained by the well synchronized cellular activities of the epithelial cells lining the epididymal mucosa. Owing to the complex functions and compartmentalisation of the epididymis, multiple causes of epididymal dysfunction resulting in male fertility disorders, and even the health of offspring, are conceivable (Chen et al., 2016a; Sharma et al., 2016; Chan et al., 2020). Defects in essential factors in the epididymis, which may originate from the testicles (Gatti et al., 1999; Shen et al., 2013; Kim et al., 2015; Koch et al., 2015; Zi et al., 2015; Liu et al., 2016), including proteins (Cornwall, 2009; Dacheux and Dacheux, 2014; Robaire and Hinton, 2015; Gervasi and Visconti, 2017), lipids (Haidl and Opper, 1997; Ouvrier et al., 2009; Saez et al., 2011; Bjorkgren et al., 2015), and non-coding RNAs (Belleannee, 2015; Chen et al., 2016a; Chen et al., 2016b; Sharma et al., 2016; Chu et al., 2017; Sharma et al., 2018), or sperm maturation deficits, impaired motility, and production of anti-sperm antibodies (Barak et al., 2000; Hamada et al., 2012), can all result in epididymal dysfunction associated male fertility deficits. Clinical practice relies on semen analysis to classify male infertility as asthenozoospermia, oligoasthenozoospermia, oligoteratozoospermia or the phenotypes associated with primitive biochemical parameters (WHO, 2021). Although male infertility is common, affecting approximately 15% of couples of child-bearing age worldwide (Zegers-Hochschild et al., 2009; Joffe, 2010; Esteves, 2013; Winters and Walsh, 2014; Tuttelmann et al., 2018; Agarwal et al., 2021), more than 50% of cases are idiopathic, i.e., of unknown cause, which limits our ability for developing targeted therapies. In some clinics before the intervention of antibiotics, epididymal deficits are estimated to be involved in 50–80% of male infertile patients (Silvert and Stanisic, 1980). Hence, an improved understanding of epididymal function permitting sperm maturation holds the promise of improved diagnosis and treatment of male infertility.

## Calcium Homeostasis and Male Reproduction

While male fertility research is predominantly focused on spermatogenesis and the biology of spermatozoa following their release from the epididymis, accumulating evidence points to epididymal function being critical for sperm activity and male fertility (Silvert and Stanisic, 1980; Chen et al., 2016b; Sharma et al., 2016; Sharma et al., 2018; Tuttelmann et al., 2018; Conine et al., 2019; Sharma, 2019; Kiyozumi et al., 2020). One unique feature of the epididymal luminal environment is its acidity and low calcium (Ca^2+^) concentration (Levine and Marsh, 1971; Levine and Kelly, 1978; Au and Wong, 1980; Jenkins et al., 1980; Hong et al., 1984; Breton et al., 1996; Clulow and Jones, 2004; Da Silva et al., 2007; Weissgerber et al., 2011). Ca^2+^ homeostasis is essential for male reproduction (Karsenty, 2011; Oury et al., 2011; Laurentino et al., 2012; Miyata et al., 2015), and Ca^2+^ dysregulation is associated with male infertility (Okunade et al., 2004; Prasad et al., 2004; Brandenburger et al., 2011; Weissgerber et al., 2011), although the mechanisms regulating Ca^2+^ homeostasis remain largely unknown. In principle, Ca^2+^ homeostasis in male reproduction organs can be regulated through inter-organ and intra-organ mechanisms, in a manner of endocrine (Karsenty, 2011; Oury et al., 2011; Oury et al., 2013), paracrine (Gao da et al., 2016; Ma et al., 2019), or lumicrine (Laurentino et al., 2012; Kiyozumi et al., 2020), and so might affect epididymal function directly or indirectly (Lewis and Aitken, 2001; Ecroyd et al., 2004a; Miyata et al., 2015).

Ca^2+^ homeostasis requires a balance of Ca^2+^ efflux and influx to maintain intracellular and extracellular Ca^2+^ concentrations within optimal ranges in individual compartments of biological systems. The proteins and mechanisms underlying Ca^2+^ homeostasis are tightly regulated. Importantly, Ca^2+^ servers as an extracellular first messenger and intracellular second messenger in numerous physiological functions (Peng et al., 2003; Hoenderop et al., 2005; Breitwieser, 2008; Bagur and Hajnoczky, 2017). In the human body, Ca^2+^ is stored in bones and teeth, mainly in the form of hydroxyapatite and Ca^2+^ phosphate. In blood plasma, circulating throughout the body, [Ca^2+^] is controlled approximately 2.5 mM; whereas in the epididymis the Ca^2+^ concentration ranges from approximately 1.3 mM at the initial segment down to 0.25 mM in the cauda (Jenkins et al., 1980; Turner, 1991; Clulow et al., 1994; Turner, 2002; Carlson et al., 2003; Carlson et al., 2007; Ma et al., 2019). Understanding the mechanisms for Ca^2+^ regulation in the epididymis will provide insights into sperm physiology and associated functions of sperm fertilization, as well as male reproductive health.

As in other systems, Ca^2+^ homeostasis in the epididymis is strictly regulated by a network of cell-cell interactions and signaling pathways. The consequences of dysregulated Ca^2+^ levels and Ca^2+^-regulated proteins in the epididymis associated with male infertility are considered as interrupting this network (Okunade et al., 2004; Schuh et al., 2004; Weissgerber et al., 2011; Correia et al., 2013). In this review, we will focus on the Ca^2+^ homeostatic regulation mechanisms in the epididymis and discuss their physiological roles in regulating the luminal fluid microenvironment for sperm maturation. Specifically, the potential role of Ca^2+^ as a cofactor for matrix Gla protein (MGP)-mediated scavenging of extracellular metabolites in the epididymal microenvironment will be discussed, and the potential role of vitamins in this regard will be explored.

## Vitamins and Male Reproduction

Whereas the essential roles of vitamins in general biology are well recognized, their specific roles in male reproductive health are remained incompletely understood. It has been reported that specific vitamin supplements can increase sperm quality in rats (Dawson et al., 1992; Wong W. Y. et al., 2002; Paradiso Galatioto et al., 2008; Blomberg Jensen et al., 2011), demonstrating that vitamins contribute to male fertility. Functional studies have revealed that vitamins A and B12 are involved in spermatogenesis (Watanabe et al., 2007; Raverdeau et al., 2012; Boucheron-Houston et al., 2013), whereas the antioxidant properties of vitamins C and E are believed to protect sperm DNA (Dawson et al., 1992; Greco et al., 2005). Vitamin B12 deficiency during maternal pregnancy or during growth of male rats was found to cause irreversible damage to the development of germ cells in embryos and affect the maturation of spermatozoa (Watanabe et al., 2003; Watanabe et al., 2007).

It is known that vitamins interact with Ca^2+^ homeostasis pathways. For example, vitamin B6 deficiency in rats was found to alter intracellular Ca^2+^-homeostasis in enterocytes, potentially via Ca^2+^ channel modulation without affecting net Ca^2+^ transport (Lal and Dakshinamurti, 1993; Matyaszczyk et al., 1993). Moreover, vitamin C can enhance intestinal Ca^2+^ absorption (Morcos et al., 1976), potentially by modulating epithelial transcellular and/or paracellular transport pathways. Vitamin C deficiency is related to secondary hyperparathyroidism in renal disease (Richter et al., 2008), and parathyroid hormone is an important endocrine signaling pathway for Ca^2+^-homeostasis (Lee and Partridge, 2009; Andrukhova et al., 2016). It is well-known that vitamin D plays an indispensable role in Ca^2+^ homeostasis regulation, possibly through the steroid sex hormone-related pathways and calcium-sensing receptor (Blomberg Jensen et al., 2010; Blomberg Jensen et al., 2011; Blomberg Jensen, 2014; Boisen et al., 2021). Our research on Ca^2+^ reabsorption in the rat epididymis pointed to the activity of transient receptor potential vanilloid channel 6 (TRPV6)- and transmembrane protein 16A (TMEM16A, also known as anoctamin-1)-associated activities in Ca^2+^- and fluid-homeostasis (Gao da et al., 2016), a pathway that has also been reported to be regulated by vitamin D (Walters et al., 2006) and that might be of fundamental importance for vital events (Rock et al., 2008; Benedetto et al., 2019), in addition to male fertility (Weissgerber et al., 2011; Weissgerber et al., 2012; Boisen et al., 2021). Vitamins D and E are lipid-soluble antioxidants and known to have protective effects on sperm quality and DNA integrity in rats challenged with oxidative stress (Greco et al., 2005; Momeni and Eskandari, 2012). While vitamin E administration alone in men of infertile couples did not improve sperm parameters upon conventional sperm analysis (Matorras et al., 2020), it did so when used in combination with selenium (Keskes-Ammar et al., 2003). Regarding vitamin K, the endocrine function of the vitamin K-dependent Ca^2+^-binding protein osteocalcin in male reproduction has been reported (Karsenty, 2011; Oury et al., 2011; Oury et al., 2013; Patti et al., 2013). Using a rat model of warfarin-induced vitamin K2-deficiency, we also confirmed the role of vitamin K-dependent MGP carboxylation in Ca^2+^-homeostasis, sperm maturation and male fertility (Ma et al., 2019). In this review, we will also discuss the potential role of vitamin interactions on epididymal calcium homeostasis, especially in relation to carboxylated MGP-mediated function.

## Epididymal Epithelial Cells and Luminal Microenvironment Are Key Players of Male Reproduction

The mammalian epididymis is a single, long, and highly convoluted tubule that connects the efferent ducts from the testis to the vas deferens. Based on morphology and regional gene expression, the epididymis is divided into four main regions, namely the initial segment and the caput, corpus and cauda epididymidis (Figure 1B). The epididymal tubule is lined with an epithelium composed of a pseudo-stratified layer of specialized epithelial cells, including principal cells, narrow cells, clear cells, basal cells and immunological cells (Da Silva et al., 2011; Robaire and Hinton, 2015; Breton et al., 2019; Rinaldi et al., 2020). These epithelial cells have cell-to-cell contact with spermatozoa, create and maintain the epididymal luminal microenvironment, including secreting maturation-promoting factors and conveying the environmental factors to the spermatozoa, as well as removing potentially harmful metabolites from the lumen, and thus play a critical role in regulation of male fertility and even the health of offspring (Robaire and Hinton, 2015; Chen et al., 2016a; Chen et al., 2016b; Sharma et al., 2016; Gervasi and Visconti, 2017; Conine et al., 2018; Sharma et al., 2018; Sharma, 2019). Understanding the essential ingredients and optimal composition of the epididymal microenvironment will improve our understanding of sperm maturation and of sperm function in the female genital tract to required to fertilize an oocyte successfully.

### Epididymal Epithelial Cells and Cell-Cell Crosstalk

**FIGURE 2:**
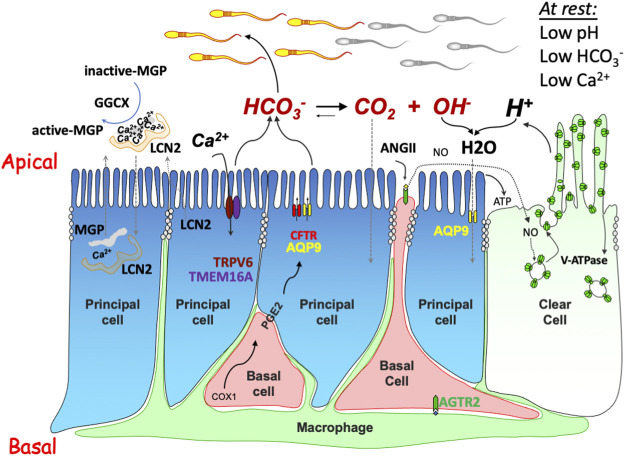
Schematic drawing showing the intercellular communication model regulating the luminal microenvironment in the epididymis. The model includes several physiological processes: 1) anion secretion (e.g., HCO_3_^−^) via apically located CFTR (and/or other anion channels, such as TMEM16A) provides an ionic gradient which drives fluid transport through water channels, e.g., aquaporin 9 (AQP9); 2) proton-pump V-ATPase acidifies the luminal content and promotes HCO_3_^−^ hydrolysis for water and CO_2_ absorption, an essential acidification process required for maintaining sperm in a quiescent state and to prevent premature activation. The acidification process is mediated by either constitutively by recycling of the V-ATPases which are abundantly expressed in clear cells, or by the stimulation of luminal factors, such as ATP generated by principal cells or nitric oxide (NO) from the basal cells upon stimulation by the angiotensin receptor type 2 (AGTR2); and 3) Ca^2+^ reabsorption through either the epithelial Ca^2+^ channel TRPV6 and Ca^2+^-dependent Cl^−^-channel TMEM16A electrical coupler or via a process facilitated by GGCX-mediated carboxylation-dependent activation of MGP for Ca^2+^ chelation and simultaneous protein aggregation, such as lipocalin 2 (LCN2).

Spermatozoa depend on the proper function of various types of epididymal epithelial cells during their transit (Battistone et al., 2020; Breton et al., 2019; Cheung et al., 2005; Leung et al., 2004; Ma et al., 2019; Robaire and Hinton, 2015; Wong P. Y. D. et al., 2002). The epithelial cells form a barrier that separates the luminal cavity from the bloodstream, and also transport nutrients and metabolites across it. Therefore, both secretory and resorptive epithelial machinery acts to create a special milieu in the lumen of the epididymis that is required for sperm maturation, while keeping them quiescent, preventing premature activation (Carr and Acott, 1984; Robaire and Hinton, 2015; Zhou et al., 2004). Epididymal function involves a network of complex regulatory mechanisms that are not yet fully understood. Using state-of-the-art tools, such as single-cell omics and systems biology analyses, recent studies have revealed the complexity of cell biology in epididymal function (de Lima et al., 2021; Rinaldi et al., 2020; Shi et al., 2021). A diagram of a basic model for the epithelial function, regulation of luminal microenvironment, and cell-cell interactions in the epididymis is presented in Figure 2. It has reported that the basal cells in the epididymis participate an active role in regulating the principal cell-mediated fluid secretion into the lumen using the prostaglandin-E2 (PGE_2_) signaling axis from the basolateral side (Leung et al., 2004; Cheung et al., 2005). In addition to this paradigm that basal cells solely play their role in the basolateral side, our research has also revealed that basal cells also have an important role from the luminal side, by sending out the antenna-like body projections to sense luminal hormonal factors, in particular angiotensin II (Shum et al., 2008). These basal cells express angiotensin type-2 receptor (AGTR2) and communicate their findings using the nitric oxide (NO) and soluble guanylyl cyclase signaling axis to the adjacent clear cells, which express high levels of proton pump vacuolar-ATPase (V-ATPase) and thereby modulate the luminal acidification (Breton and Brown, 2013), an essential process for sperm maturation and viability (Robaire and Hinton, 2015). The cell-cell interactions between clear cells and principal cells, between principal cells, as well as between macrophages and epithelial cells have also been described (Battistone et al., 2019; Breton et al., 2019; Battistone et al., 2020).

The epididymal epithelium is known to be active in secretion and absorption via the sophisticated exocytotic and endocytotic cellular machinery, predominantly in the principal cells and clear cells (Leung et al., 2004; Cheung et al., 2005; Robaire and Hinton, 2015; Gao da et al., 2016; Ma et al., 2019). These are the major cell types covering most of the epididymal luminal surface and thus mainly responsible for the controlling luminal ion compositions, luminal organic molecules, and liberating extracellular vesicles (EVs), also known as epididymosomes. These membraned-bound EVs serve as the key extracellular medium for two-way cell-to-cell communication between the nurtured spermatozoa and the nurturing epididymal cells (Sullivan et al., 2005; Sullivan et al., 2007; Sharma et al., 2016; Conine et al., 2018; Sharma et al., 2018; Leahy et al., 2020).

Other cell types including the basal cells and the immunological cells like dendritic cells can occasionally make contact to the luminal environment in a regional-dependent manner (Shum et al., 2008; Da Silva et al., 2011; Shum et al., 2014). Their contribution to the luminal environment is thought to be indirect and cell-cell interactions are the underlying indispensable mechanisms (Shum et al., 2008; Da Silva et al., 2011; Shum et al., 2014; Battistone et al., 2020). It is reasonable to hypothesize that the luminal microenvironment in epididymis critically affects sperm function, and thus male fertility and even transgenerational health of offspring (Chen et al., 2016a; Chen et al., 2016b; Sharma et al., 2016; Conine et al., 2018; Sharma, 2019; Chan et al., 2020).

### The Composition of Epididymal Luminal Fluid

The epididymal luminal fluid, similar to other bodily extracellular fluids, comprises cations and anions, with organics constituents of proteins and other macromolecules and small molecules, glycans and lipids (Pastor-Soler et al., 2005; Cornwall, 2009; Shum et al., 2011; Robaire and Hinton, 2015; Tecle and Gagneux, 2015; Gervasi and Visconti, 2017; Zhou et al., 2018; Cornwall et al., 2019). The ionic composition together with the organic matter determines the fluid volume, pH and osmolarity. Before entering the epididymal tubule, immature spermatozoa in testicular fluid enter the efferent ducts (Wong and Yeung, 1978; Wong P. Y. D. et al., 2002), where the majority (>95%) of the fluid is reabsorbed (Hess et al., 1997; Clulow et al., 1998; Wong P. Y. D. et al., 2002; Turner, 2002), and replaced with epididymal transcellular fluid, the fluid within epithelial-lined space. Upon transit from the head down to the tail of epididymis, addition >90% of epididymal transcellular fluid is reabsorbed (Yamamoto et al., 1993; Wong P. Y. D. et al., 2002; Turner, 2002).

#### The Epididymal Luminal Fluid Is a Matrix With Regional Specificity

Epididymal fluid is a gel-like and viscous dynamic phase compartment, which can be characterized as a giant membraneless organelle (Cornwall, 2009; Shum et al., 2011; Robaire and Hinton, 2015; Tecle and Gagneux, 2015; Gervasi and Visconti, 2017; Zhou et al., 2018; Cornwall et al., 2019). The intraluminal fluidic contents are compartmentalized distinctly according to anatomical regions of the epididymal tubule, as revealed by a glance at the region-specific patterns of different types of epithelial cells and their absorption and secretory machinery, as well as the changes of surface contents on the surface of epididymal spermatozoa (Turner et al., 2003; Ecroyd et al., 2004b; Johnston et al., 2005; Pastor-Soler et al., 2005; Yudin et al., 2005; Finger et al., 2006; Jelinsky et al., 2007; Turner et al., 2007; Shum et al., 2011; Tollner et al., 2012; Shum et al., 2014; Robaire and Hinton, 2015; Zhou et al., 2018). The mechanisms underlying the formation and maintenance of epididymal fluid compartmentalization are not fully understood yet. However, liquid-liquid phase separation, a widespread mechanism for cells to handle membraneless matrices (Hyman et al., 2014; Shin and Brangwynne, 2017) suggest that this process could play a role in regulation of epididymal luminal microenvironment.

#### Water and Acid-Base Balance

The concentrations of all the epididymal fluid components reflect their dilution in water, whose volume in turn is adjusted as a function of the bathed ion concentrations; thus water transport is important for the optimal microenvironment in each compartment of the male excurrent duct, ensuring sperm maturation. And thus, water transport also influences Ca^2+^ homeostasis in the epididymal lumen, despite the regulation mechanism and the physiological implications still largely remain to explore. Proteins known to be relevant for water transport and electrolyte balance in epididymis, including cystic fibrosis transmembrane conductance regulator (CFTR) (Wong, 1998; Ruan et al., 2012), aquaporins (Stevens et al., 2000; Pastor-Soler et al., 2001; Cheung et al., 2003; Pastor-Soler et al., 2005; Da Silva et al., 2006), and the Na^+^-K^+^-pump and associated transporters (Breton et al., 1998; Bagnis et al., 2001; Wong P. Y. D. et al., 2002; Kujala et al., 2007; Zuo et al., 2011), have important role in maintaining epididymal function and male fertility (Wagenfeld et al., 2002; Xu et al., 2003; Yeung et al., 2004; Pruneda et al., 2007; Krausz and Riera-Escamilla, 2018; Xu et al., 2018).

Water is the medium of numerous hydrolysis processes in biological systems and carbon dioxide and its hydrolyzed derivative bicarbonate are not only the metabolic products but also an acid-base balancers in the body. In epididymis, bicarbonate-related acid-base balance is an essential process for acidification of luminal fluid, which is critical for maintaining spermatozoa in a quiescent state (Au and Wong, 1980; Carr and Acott, 1984; Breton et al., 1996; Newcombe et al., 2000; Pastor-Soler et al., 2005; Shum et al., 2011; Zuo et al., 2011). Hence, multitude of proteins must act in concert to establish and maintain optimal epididymal volume, electrolyte balance, and pH. The orchestration of these multiple proteins and their functions involves a complex network of cell-cell crosstalk involving autocrine, paracrine and lumicrine signaling (Kim et al., 2015; Robaire and Hinton, 2015; Gao da et al., 2016; Battistone et al., 2019; Ma et al., 2019; Kiyozumi et al., 2020). Figure 2 shows a basic model for the epithelial cell functions involved in epididymal fluid regulation, including water and ion transport, acid-base balance, and calcium homeostasis.

#### Inorganic Ions in the Epididymis

Table 1 summarises the concentrations of most inorganic ions contained in epididymal fluid, based on data published in the literature (Levine and Marsh, 1971, 1975; Levine and Kelly, 1978; Hinton and Setchell, 1980; Jenkins et al., 1980; Turner et al., 1984; Caflisch and DuBose, 1990; Hinton, 1990; Caflisch, 1992; Cooper et al., 1992; Clulow et al., 1994; Stoltenberg et al., 1996; Clulow et al., 1998; Sorensen et al., 1999; Newcombe et al., 2000). The high K^+^ ion concentration, up to approximately 55 mM in the distal cauda region (approximately ten-fold higher than in serum), and the low concentration of Ca^2+^ (down to 0.25 mM in the cauda region, approximately ten-fold lower than in serum) are notable. These special properties indicate that the microenvironment for sperm maturation is uniquely formed and maintained as spermatozoa transit the epididymal tubule.

**TABLE 1:** Concentrations of inorganic elements (mM) and pH in blood plasma and intraluminal fluids from the excurrent duct of rats.

	Blood	Seminiferous tubule (SNT)	Rete testis	Efferent duct	Initial segment	Caput	Corpus	Cauda	Vas deferens
Na^+^	138.65∼147.2^a^ ^,^ ^b^ ^,^ ^c^	109.5∼135.44^a^ ^,^ ^b^	130.8∼141.84^b^ ^,^ ^c^ ^,^ ^d^	144.2^c^	136.8^c^	101.8∼112.1^a^ ^,^ ^b^ ^,^ ^d^	57.9∼93.8^a^ ^,^ ^b^	20.6∼37.17^a^ ^,^ ^b^ ^,^ ^d^	23.3^a^
K^+^	4.9∼5.83^b^ ^,^ ^c^	39.77∼46.2^a^ ^,^ ^b^	12.4∼16.1^b^ ^,^ ^c^ ^,^ ^d^	5.7^c^	11.6^c^	16.0∼27.6^a^ ^,^ ^b^ ^,^ ^d^	37.3∼38.3^a^ ^,^ ^b^	39.98∼55.1^a^ ^,^ ^b^ ^,^ ^d^	51.9^a^
Ca^2+^	0.52∼2.4^b^ ^,^ ^c^	0.44^b^	0.81∼0.9^b^ ^,^ ^c^	2.2^c^	1.3^c^	0.85^b^	0.51^b^	0.25^b^	?
Mg^2+^	0.37∼3.3^b^ ^,^ ^c^	1.19^b^	0.39∼1.5^b^ ^,^ ^c^	2.7^c^	1.7^c^	1.97^b^	2.61^b^	0.9^b^	?
Cl^−^	98.0∼122.14^a^ ^,^ ^b^ ^,^ ^c^	118.0∼143.37^a^ ^,^ ^b^	129.7∼135.76^b^ ^,^ ^c^	112.8^c^	116.7^c^	24.25∼31.0^a^ ^,^ ^b^	24.4∼39.09^a^ ^,^ ^b^	23.6∼27.04^a^ ^,^ ^b^	19.3^a^
Total P^e^	2.25∼3.5^b^ ^,^ ^c^	9.22^b^	1.2∼1.72^b^ ^,^ ^c^	3.2^c^	4.5^c^	59.22∼82.5^b^ ^,^ ^f^	80.8∼93.76^b^ ^,^ ^f^	79.4∼88.7^b^ ^,^ ^f^	73.3^f^
HCO_3_^−^	23.0∼30.1^a^ ^,^ ^g^ ^,^ ^h^	10.6∼19^a^ ^,^ ^g^	22.9^h^	45.2^h^	8.7∼20.4^g^ ^,^ ^h^	2.7∼4.8^a^ ^,^ ^g^	?	6.7^a^	6.7^a^
pH	7.39∼7.5^a^ ^,^ ^g^ ^,^ ^h^ ^,^ ^i^	6.93∼7.31^a^ ^,^ ^g^ ^,^ ^i^ ^,^ ^j^	7.34^h^	7.66^h^	6.79∼7.26^g^ ^,^ ^h^ ^,^ ^j^	6.48∼6.64^a^ ^,^ ^g^ ^,^ ^i^ ^,^ ^j^	7.10∼7.18^g^ ^,^ ^i^	6.85∼6.88^a^ ^,^ ^g^ ^,^ ^i^	6.85^a^
Osmolarity^k^	299.4∼311^a^ ^,^ ^c^	338^a^	306.6^c^	303.1^c^	300.5^c^	315^a^	340^a^	329^a^	339^a^

a Data included from **Levine and Marsh (1971)**.

b Data included from **Jenkins et al. (1980)**.

c Data included from **Clulow et al. (1994)**.

d Data included from **Turner (1984)**.

f Data included from **Hinton and Setchell (1980)**.

g Data included from **Caflisch (1992)**.

h Data included from **Newcombe et al. (2000)**.

i Data included from **Caflisch and DuBose (1990)**.

j Data included from **Levine and Kelly (1978)**.

e Total P represents measuremetns including inorganic phosphorus, glycerophosphocholine and phosphocholine.

k Osmolarity unit: mOsm/kg.

## Calcium Homeostasis in the Epididymal Luminal Matrix

### The Epididymis Maintains a Low Luminal Ca^2+^ Microenvironment

After spermatozoa are released from the testis, they migrate passively towards the excurrent duct and enter the epididymal tubule where they mature along a spatio-temporally changing milieu. As mentioned earlier, >90% of the initial epididymal fluid is reabsorbed by the epididymal epithelium during its transit toward the cauda. Therefore, it is suggestive that the low concentration of Ca^2+^ in the cauda depend on active Ca^2+^ reabsorption machinery in the epididymis. Consistent with the critical role of a low Ca^2+^ concentration in the epididymal fluid, dysregulation of epididymal Ca^2+^ homeostasis is one cause of male infertility (Prasad et al., 2004; Schuh et al., 2004; Brandenburger et al., 2011; Karsenty, 2011; Oury et al., 2011; Weissgerber et al., 2011; Laurentino et al., 2012; Oury et al., 2013; Miyata et al., 2015).

In general, epithelial absorption can occur through two main pathways for ion transport across epithelial cells: one is the paracellular pathway, in which electrochemical gradients passively drive ions through tight junctions and the paracellular space; the other is the transcellular pathway, which involves several steps including active apical absorption and intracellular trafficking as well as basolateral secretion (Hoenderop et al., 2005; Frizzell and Hanrahan, 2012; Garcia-Castillo et al., 2017). Because the transepithelial Ca^2+^ gradient does not support passive Ca^2+^ absorption through the paracellular pathway, transcellular Ca^2+^ absorption is expected the main pathway of Ca^2+^ absorption in the epididymis.

The transcellular transport of Ca^2+^ across epithelia is a multistep process (Hoenderop et al., 2005), beginning with passive entry of Ca^2+^ through the Ca^2+^ channels in the apical membranes followed by diffusion through cytosol facilitated by binding to intracellular Ca^2+^-binding proteins, e.g. calbindin-D28k in the kidney and calbindin-D9K in the intestine (Hoenderop et al., 2005), and eventually extrusion across the basolateral membranes by energy-requiring Na^+^/Ca^2+^ exchangers, plasma membrane Ca^2+^-ATPases (PMCAs), which operate against the electrochemical gradient for Ca^2+^ (Okunade et al., 2004; Hoenderop et al., 2005; Brandenburger et al., 2011; Patel et al., 2012). From a stoichiometric point of view, initial step of passive entry through apical Ca^2+^ channels is believed to be likely the rate-limiting step of the transepithelial Ca^2+^ (Nijenhuis et al., 2005). In epithelial tissues, only two highly Ca^2+^-selective ion channels act as apical channels for Ca^2+^ (re)absorption: Ca^2+^-selective ion channels TRPV5 and TRPV6 (Peng et al., 1999; Peng et al., 2000). Our previous study found that only TRPV6 was expressed in the apical pole of the rat epididymal epithelium of rats in a regional-dependent manner (Shum et al., 2006). Studies from other research groups using genetic deletion or mutant mouse models of the epithelial calcium channel TRPV6 have confirmed that apical Ca^2+^ influx through TRPV6 is important for the prevention of abnormal Ca^2+^ accumulation in the epididymis (Weissgerber et al., 2011; Weissgerber et al., 2012). In these studies, it was shown that after the ablation of TRPV6 channel function, the Ca^2+^ concentration in the distal cauda epididymidis was increased nearly ten-fold, sperm motility decreased, and fertilizing ability in mice was reduced. These studies showed that the epididymal Ca^2+^ homeostasis is critical for male fertility and sperm maturation.

Other mechanisms of epididymal Ca^2+^ homeostasis are also critical for male fertility and post-epididymal sperm functions. For example, in the plasma membrane Ca^2+^-pump 4 (PMCA4) knockout mice, sperm lacked motility and could not move forward in the female reproductive tract, resulting in male infertility (Okunade et al., 2004; Brandenburger et al., 2011). PMCA4a protein may be transported via epididymosomes from the epithelial cells to the sperm tail in the epididymis (Brandenburger et al., 2011), a process regulated by luminal constituents. Another observation pointing to the importance of Ca^2+^ homeostasis for male fertility is that loss of the Ca^2+^-dependent phosphatase calcineurin leads to infertility in male mice, owing to decreased sperm motility caused by an inflexible midpiece of spermatozoa (Miyata et al., 2015). Moreover, the functionality for sperm capacitation, which is triggered by Ca^2+^ influx in spermatozoa with subsequent activation of the cAMP-PKA signaling pathway and protein tyrosine phosphorylation, is formed in the epididymis and sensitive to extracellular Ca^2+^ (Luconi et al., 1996; Lewis and Aitken, 2001; Ecroyd et al., 2005; Gervasi and Visconti, 2016; Puga Molina et al., 2017; Takei et al., 2021). Another Ca^2+^-binding protein regucalcin, is abundant in epididymal luminal fluid, but any role in Ca^2+^ homeostasis remains to be demonstrated (Laurentino et al., 2012; Correia et al., 2013). Taken together, these findings point to the complexity and importance of Ca^2+^ homeostasis in the lumen of epididymis.

### Regulation of Low Ca^2+^ Concentrations in the Epididymis and Its Molecular Mechanisms and Physiological Implications

It has been shown that defects in transepithelial Ca^2+^ absorption, including by defects of the apically located TRPV6 Ca^2+^ channels or the basolateral PMCA4 Ca^2+^ exclusion pump, result in Ca^2+^ accumulation in the luminal compartment of the distal epididymal tubule. This in turn impairs epididymal-dependent spermatozoa fertilization activities and impaired fertility without affecting spermatogenesis (Okunade et al., 2004; Schuh et al., 2004; Weissgerber et al., 2011; Weissgerber et al., 2012; Patel et al., 2013).

#### Regulating Epididymal Ca^2+^ Homeostasis by the TRPV6-TMEM16A Coupler

In further characterization of the mechanisms regulating Ca^2+^ absorption in the epididymis, our research has demonstrated that the TRPV6-associated extracellular pH-sensitive Ca^2+^ conductance functions in an electrically coupled manner with the intracellular Ca^2+^-sensitive chloride channel TMEM16A in isolated single principal cells of the distal part of the rat epididymis (Gao da et al., 2016) (see Figure 2). The interplay between TRPV6 and TMEM16A does not only regulate Ca^2+^ resorption but also Ca^2+^-dependent anionic current, a driving force of fluid secretion (Wong P. Y. D. et al., 2002). Our research also found that TRPV6-like Ca^2+^ conductivity is regulated by negative membrane potential and is sensitive to extracellular pH and divalent ions (Ca^2+^ and Mg^2+^), all of these physiological features are uniquely formed and maintained in the epididymis. Therefore, the coupling of TRPV6 with TMEM16A makes this coupler a key player of epididymal secretion. TMEM16A has been described to mediate epithelial mucin secretion, including in response to inflammation (Huang et al., 2012; Lin et al., 2015; Zhang et al., 2015; Benedetto et al., 2019). Our research has shown that TRPV6 co-localizes with TMEM16A in the apical membrane of the rat epididymal epithelium, as well as in some epididymosomes of the very proximal and the very distal rat epididymis (Gao da et al., 2016). These findings raise the possibility of TRPV6-TMEM16A contributing to the secretion of epididymosomes from the epididymal cells, an epididymal cell-to-cell communication mechanism that is essential for sperm maturation as well as transgenerational paternal epigenetic information transfer (Sullivan et al., 2005; Lotvall et al., 2014; Belleannee, 2015; Chen et al., 2016a; Chen et al., 2016b; Sharma et al., 2016; Tkach and Thery, 2016; Sharma, 2019; Chan et al., 2020).

#### Another Calcium Reabsorption Mechanism in Addition to TRPV6-TMEM16A Coupler in the Epididymis

It is known that the epididymal luminal fluid is acidic and under resting physiological conditions, it maintains a pH range of about 6.4–6.8 in the corpus and cauda epididymidis of rodents (see Table 1). Our research has shown that in single cauda epithelial cells isolated from rat epididymis, the TRPV6-related conductance is inhibited at acidic pH (for example, pH 6.4) and activated at alkaline pH (for example, 7.4) (Gao da et al., 2016). In addition, the TRPV6 activity is also sensitive to the resting membrane potential, and its conductance is inwardly rectifying at negative membrane potentials and is negligible at positive potentials (Bodding, 2005; Hoenderop et al., 2005). Whereas the resting membrane potential of epididymal epithelial cells has been determined to range between –6 mV and –39 mV, with an average value of approximately –22 mV (Cheung et al., 1976; Gao da et al., 2016). Our research has shown that in these resting potential ranges, the Ca^2+^-permeable current in single cauda epithelial cells at extracellular pH 6.4, or even at pH 7.4 is indeed undetectable or negligible, respectively (Gao da et al., 2016). We speculate that under physiological conditions of acidic epididymal lumen, Ca^2+^ influx through TRPV6 channel alone is silent or at least negligible, while the electrical coupling between TRPV6 and TMEM16A promotes Ca^2+^ cation influx by accompanying Cl^−^ anion influx. However, the restricted localization of the extracellular pH- and Ca^2+^-dependent TRPV6-TMEM16A electrical coupler in the proximal and distal ends of the epididymis as well as in the epididymosomes not associated with extensive Ca^2+^ reabsorption could imply the presence of another calcium reabsorption mechanism or that TRPV6 and its coupler TMEM16A have a function other than Ca^2+^ reabsorption. Consistent with our speculation, a study of roosters found that those with epididymal stones had raised epididymal expression of TRPV6, although the connection between the two observations is still unclear (Oliveira et al., 2012).

#### Vitamin K2-Dependent Matrix Gla Protein-Mediated Ca^2+^-Homeostatic Regulation and Its Role in Epididymal Luminal Microenvironment and Male Fertility

To address whether in epididymidal regions where TRPV6-TMEM16A is absent, another Ca^2+^ reabsorption mechanism is active, our research has found vitamin K-dependent γ-glutamyl carboxylase (γ-carboxylase or GGCX)-mediated carboxylation of the substrate MGP to play a role in Ca^2+^ homeostasis in the epididymis (Ma et al., 2019). Consistent with our hypothesis, we found that GGCX-MGP expression was enriched in the epididymal regions with minimal TRPV6-TMEM16A coupler expression. Using a rat model of warfarin-induced vitamin K2 deficiency, we found that vitamin K2-dependent GGCX and its γ-carboxylation substrate MGP were essential for epididymal Ca^2+^ homeostasis, sperm maturation and thereby male fertility. Warfarin-induced vitamin K2-deficiency in rats leads to disruption of GGCX-mediated carboxylation of MGP, which leads to accumulation of stress granules and Ca^2+^ in the epididymal epithelium and lumen, and ultimately reduces male fertility (Ma et al., 2019).

#### Insights Into the Role of Carboxylation-Dependent Calcium Homeostasis in the Epididymis on Male Reproduction and Offspring Health

Vitamin K is a known key modulator of Ca^2+^ homeostasis. By facilitating GGCX-mediated γ-carboxylation of matrix proteins, including MGP, it maintains bone health and prevents vascular ectopic calcification (Weber, 2001; Wallin et al., 2008; Boraldi et al., 2013). MGP is a 14-kDa (103 amino acids) secretory protein containing five Ca^2+^-binding γ-carboxyglutamic acid (Gla) residues. It was first described as an extrahepatic matrix protein in the extracellular matrix of bone and cartilage and later detected in a wide variety of other tissues such as lung, heart, kidney and arterial vessel walls (Price et al., 1983; Fraser and Price, 1988; Hale et al., 1988). MGP is synthesized locally, at least in the vascular tissues, where it counterbalances ectopic mineral deposition (Murshed et al., 2004). The essential role of MGP has been revealed in mice lacking MGP, which die within 6–8 weeks of birth from large blood vessels ruptures, owing to massive vascular calcification (Luo et al., 1997). Humans with mutations in both alleles of the *MGP* gene develop Keutel syndrome, an autosomal recessive disorder characterized by abnormal calcification of soft tissues and female miscarriages (Munroe et al., 1999; Khosroshahi et al., 2014). The vitamin K antagonist, warfarin through inhibition of the vitamin K epoxide reductase complex 1 (VKORC1) to prevent carboxylation and activation of MGP. The treatment of normal mice with warfarin has been associated with a rapid calcification of elastic lamellae in arteries and heart valves that is reminiscent of the MGP-null phenotype in mice (Price et al., 1998). The importance of vitamin K status for MGP has also been confirmed in healthy people and in patients with long-term warfarin treatment (Rennenberg et al., 2010; Cranenburg et al., 2012). Importantly, the understanding of vitamin K-dependent mechanisms for local calcification regulation may also provide information about luminal calcium homeostasis in the epididymis.

Like other Gla proteins, MGP is activated by GGCX, which is the only gamma-carboxylation enzyme in the cell and which has no relevant homology to any known enzyme families (Tie et al., 2016). The Ca^2+^ binding function of MGP requires two post-translational modifications: γ-glutamate carboxylation and serine phosphorylation (Schurgers et al., 2007; Schurgers et al., 2013; Houben et al., 2016). The exact mechanism through which serine phosphorylation in MGP facilitates Ca^2+^ binding is unclear, and may involve indirect mechanisms such as promoting extracellular secretion of MGP (Wajih et al., 2004). In contrast, γ-glutamyl carboxylation is precisely related to increased Ca^2+^ binding affinity of MGP (Hackeng et al., 2001; Schurgers et al., 2005).

Our research has showed that MGP expression is high in the epididymis when compared with other organs containing epithelial-lining such as kidney and liver (Ma et al., 2019). In addition, GGCX and MGP co-localized in the vesicular structures on the luminal surface of epithelial and sperm membranes, potentially indicating that carboxylation takes place in the epididymal luminal microenvironment. These results may dicates that the vitamin K-dependent carboxylation and the MGP-associated prevention of Ca^2+^-deposition can reciprocally regulate epididymal luminal secretions. Since the expression of GGCX and MGP and their co-localization on vesicular granules, sperm surface, and in the cytoplasmic droplets progressively increases from the proximal epididymis distally, our study suggests that both proteins likely arose from the epididymosomes derived from epididymal epithelial cells. In view of the important role of epididymosomes in the male reproduction and the health of offspring (Sullivan et al., 2005; Chen et al., 2016a; Chen et al., 2016b; Sharma et al., 2016; Conine et al., 2018, 2019; Sharma et al., 2018), whereas GGCX-dependent carboxylation of MGP plays a vital role in life events (e.g., early embryo development) (Schurgers et al., 2013; Khosroshahi et al., 2014), the understanding the role of carboxylated-MGP dependent luminal calcium homeostasis in epididymis may provide insights into male reproduction and the health of offspring.

### Ca^2+^ Behaves as a Cofactor of the Matrix Gla Protein-Promoted Scavenging Function of Ca^2+^-Precipitable Aggregates

#### Biphasic Ca^2+^-Dependent Chelation Properties of Matrix Gla Protein

**FIGURE 3:**
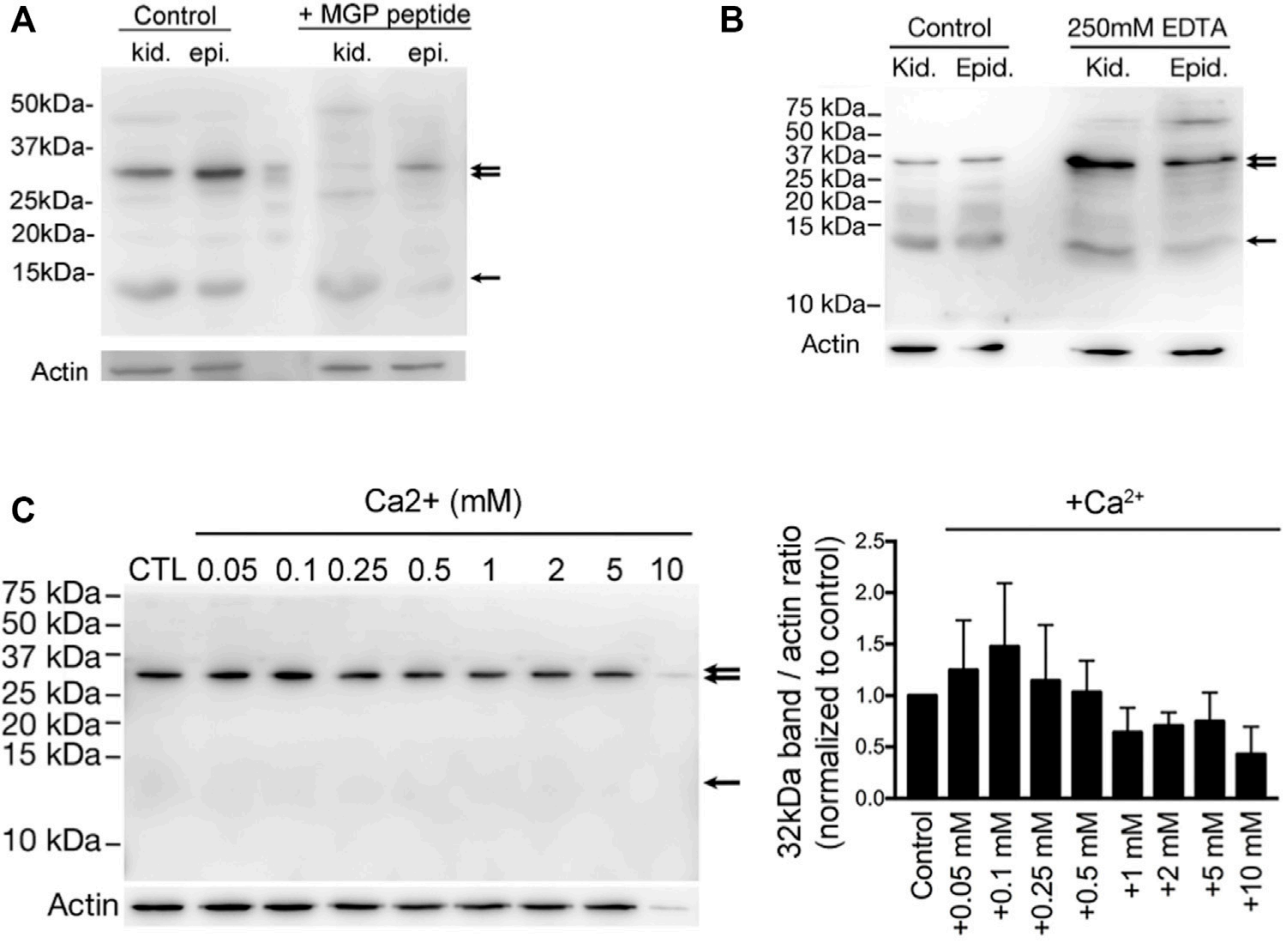
Vitamin K-dependent MGP-mediated calcium-promoted aggregation of a protein complex with a prominent band of ∼32-kDa. **(A)** Western blot detection of anti-MGP in total homogenates of kidney (kid.) and epididymis (epi.) from WT adult rats. A band at ∼12-KDa (arrow) corresponding to the expected molecular size of MGP, and another major band at around ∼32-kDa (double arrow) were detected. Both bands were almost abolished by the preincubation with a ten-fold excess of the MGP immunizing peptide (+MGP peptide). **(B)** The intensity of ∼32-kDa bands (double arrow) were significantly enriched in the low-Ca^2+^ condition by addition of 250 mM EDTA to the protein lysates, whereas the ∼12-kDa bands remained unchanged (arrow). Some bands at higher molecular sizes became obvious under the low-Ca^2+^ condition. **(C)** The same anti-MGP antibody was used to detect the intensity changes of ∼32-kDa band in DC2 cell protein lysates under various Ca^2+^ concentrations and the bar graph on the right shows the intensity of the band normalized to control (no additional Ca^2+^). This suggests that MGP-mediated protein-aggregation is dependent on sub-millimolar amount of Ca^2+^, whereas excessive Ca^2+^ (>0.25 mM) inhibits protein-aggregation. (Originally published in *iScience* (Ma et al., 2019), with permission to reproduce from *iScience*).

In one of our previous studies (Ma et al., 2019), western blotting with anti-MGP antibody on rat epididymis and kidney always detected a major band at approximately 32-kDa that did not correspond to the expected ∼12-kDa molecular weight of MGP (Figure 3). Other publications also noted this band and interpreted it as a non-specific band due to the excessive protein lysis sample (Lomashvili et al., 2011). However, the intensity of this ∼32-kDa band was strikingly decreased when the tissue protein extracts were preincubated with the MGP peptide (Figure 3A). In addition, the intensity of the 32-kDa band and a few weaker bands at higher molecular weight became intensified when the divalent ion chelator EDTA was added to the protein extracts (Figure 3B), suggesting that these bands may represent Ca^2+^-dependent protein aggregates. Supporting this hypothesis, the 32-kDa band intensity from an epididymal cells line changed in a biphasic Ca^2+^-dependent manner. When the Ca^2+^ was added to the protein extract at sub-millimolar concentrations, the intensity of the Ca^2+^-bound MGP-positive protein complex increased. Maximum intensity of the MGP-positive protein complex was observed after addition of 0.1 mM Ca^2+^, suggesting that at this concentration protein aggregation is favored. When the Ca^2+^-concentration was further increased to the millimolar range, the band intensity was reduced, presumably because millimolar Ca^2+^ does not favor formation of MGP-containing protein aggregates (Figure 3C). This Ca^2+^-dependent, biphasic protein aggregation matches the Ca^2+^ levels that have been reported for the epididymal luminal microenvironment and range from ∼0.25 to ∼0.9 mM (Jenkins et al., 1980; Turner, 1991; Clulow et al., 1994; Turner, 2002). These results suggest that MGP-containing protein aggregates form in the presence of sub-millimolar Ca^2+^, but not at millimolar Ca^2+^ concentrations.

#### Unique Properties of Matrix Gla Protein for Ca^2+^ Chelation Facilitate Its Scavenging Function in the Epididymal Lumen

**FIGURE 4:**
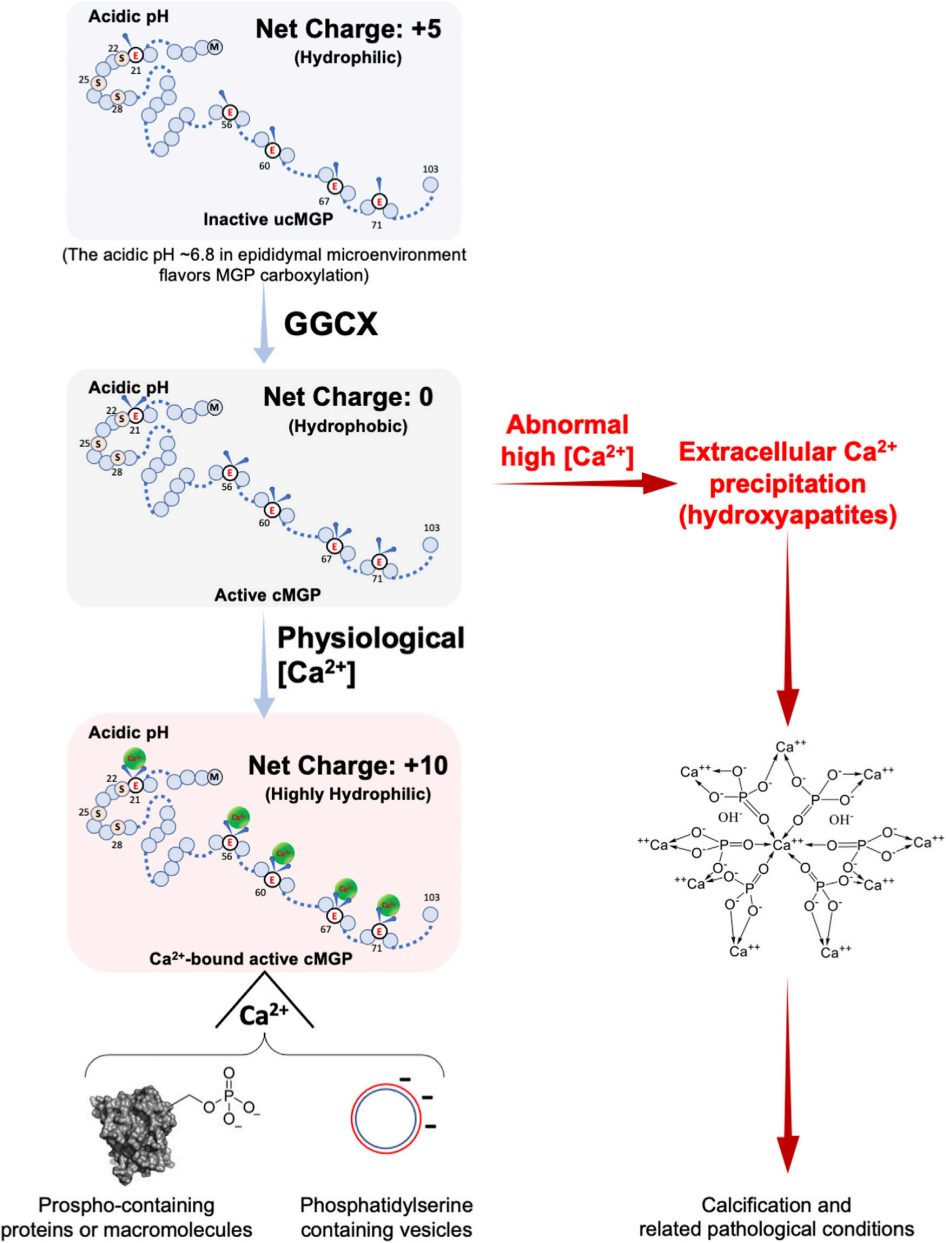
Calcium-dependent chelation property of MGP with or without carboxylation. MGP is a protein composed of 103 amino acids and can be post-translationally modified by carboxylation and phosphorylation. Uncarboxylated MGP (ucMGP) is an inactive form with an isoelectric point of around 9 (8.8 for rat and 9.6 for human MGP), which means that in an acidic environment, the ucMGP has a net positive charge of five, despite its five γ-glutamate residues, and thus it is hydrophilic. When the five γ-glutamate residues are carboxylated to γ-carboxylglutamates by GGCX enzymatic activity, the carboxylated MGP (cMGP) becomes active but is electrically neutral and thus hydrophobic. Intriguingly, the five carboxylated γ-glutamate residues of cMGP bear five negative charges, which favors the chelation of five Ca^2+^ ions and form a highly hydrophilic ten-positively-charged cMGP-Ca^2+^ compound, facilitating chelation of negative-moiety-bearing molecules under normal physiological conditions, particularly aggregation of organic or inorganic phosphates, or the membrane-embedded phosphatidylserine such as membranes of extracellular vesicles. Dysregulation of the Ca^2+^-binding property of cMGP results in ectopic Ca^2+^ precipitation and abnormal Ca^2+^ content, which causes cMGP to bind with hydroxylapatites and thereby precipitation nucleation, a precursor status of promoting matrix calcification and related pathological conditions.

The formation of the MGP-binding protein complex can be interpreted from the biochemical nature of the MGP protein itself (Figure 4). The inactive form of uncarboxylated MGP (ucMGP) protein has an isoelectric point of about 8.8, so that in the epididymal microenvironment of around pH 6.4–6.8 (Levine and Marsh, 1971; Da Silva et al., 2007), the five γ-glutamate Glu residues of ucMGP endow the protein with five positive charges and thus hydrophilic. When these Glu sites are carboxylated by GGCX, the protein becomes its active Gla form of carboxylated MGP (cMGP), in which the positive charges are neutralized, so cMPG is electrically neutral and hydrophobic. However, the carboxylated sites have strong Ca^2+^ binding ability, which permit cMGP to bind five Ca^2+^ ions (Hackeng et al., 2001; Huang et al., 2003; Schurgers et al., 2005; Boraldi et al., 2013), and thus carrying ten positive charges, greatly enhancing its solubility. Therefore, in the acidic epididymal lumen, inactive ucMGP is soluble but lacks Ca^2+^ binding ability, whereas the active cMGP is insoluble but highly adherent to Ca^2+^, which confers the aggregating ability. In other words, Ca^2+^ ions could promote Ca^2+^-MGP scavenging function. Ca^2+^-MGP chelator function could be a perfect scavenger for the aggregation of either organic or inorganic extracellular matter or both, and regulated by the changing luminal microenvironment, including nutrients supply, acid-base balance and luminal Ca^2+^ levels, as well as other constituents in epididymal fluid.

The epididymal luminal microenvironment has a complex composition and contains components that can interact with MGP other than Ca^2+^, such as Mg^2+^ and phosphorus-containing electrolytes (see Table 1). EDTA chelates divalent cations and its affinity for Mg^2+^ is higher than for Ca^2+^. The increased intensity of the MGP-positive protein aggregate bands after the addition of exogeneous EDTA to protein lysates (see Figure 3B) can be explained by preferential chelation of Mg^2+^-ions and promotion of MGP interaction with Ca^2+^ and subsequent protein aggregate formation (Ma et al., 2019). This hypothesis is consistent with the finding that Mg^2+^ and Ca^2+^ competitively bind to MGP (Roy and Nishimoto, 2002). This also indicates that under the low Ca^2+^ concentrations of the epididymal lumen, activated MGP preferentially binds to Ca^2+^ and other organic matter, as illustrated in Figure 4. In the presence of high Ca^2+^, MGP has an affinity for inorganic phosphorous compounds (Roy and Nishimoto, 2002), but this would not occur in the epididymal lumen–the microenvironment for the maturation of spermatozoa whose membrane surfaces are enriched with phosphorates in lipids and proteins (Hamamah and Gatti, 1998; Jenkins et al., 1980). Under conditions with abnormally high Ca^2+^ (see Figure 4), such as impaired GGCX activity after warfarin treatment, Ca^2+^ precipitated granules result (Ma et al., 2019) and other pathological conditions may occur (Tan et al., 2012). A related phenomenon may be the clinical observations that patients undergoing continuous hemodialysis have a higher incidence of epididymal stones (Guvel et al., 2004; Bozzini et al., 2013), although the correlation and mechanism between these two phenomena are still unknown.

#### Insights Into the Mechanism Linking the GGCX arginine^325^ to glutamine^325^ (R325Q) rs699664 Polymorphism With the Increased Infertility Risk in Men

**FIGURE 5:**
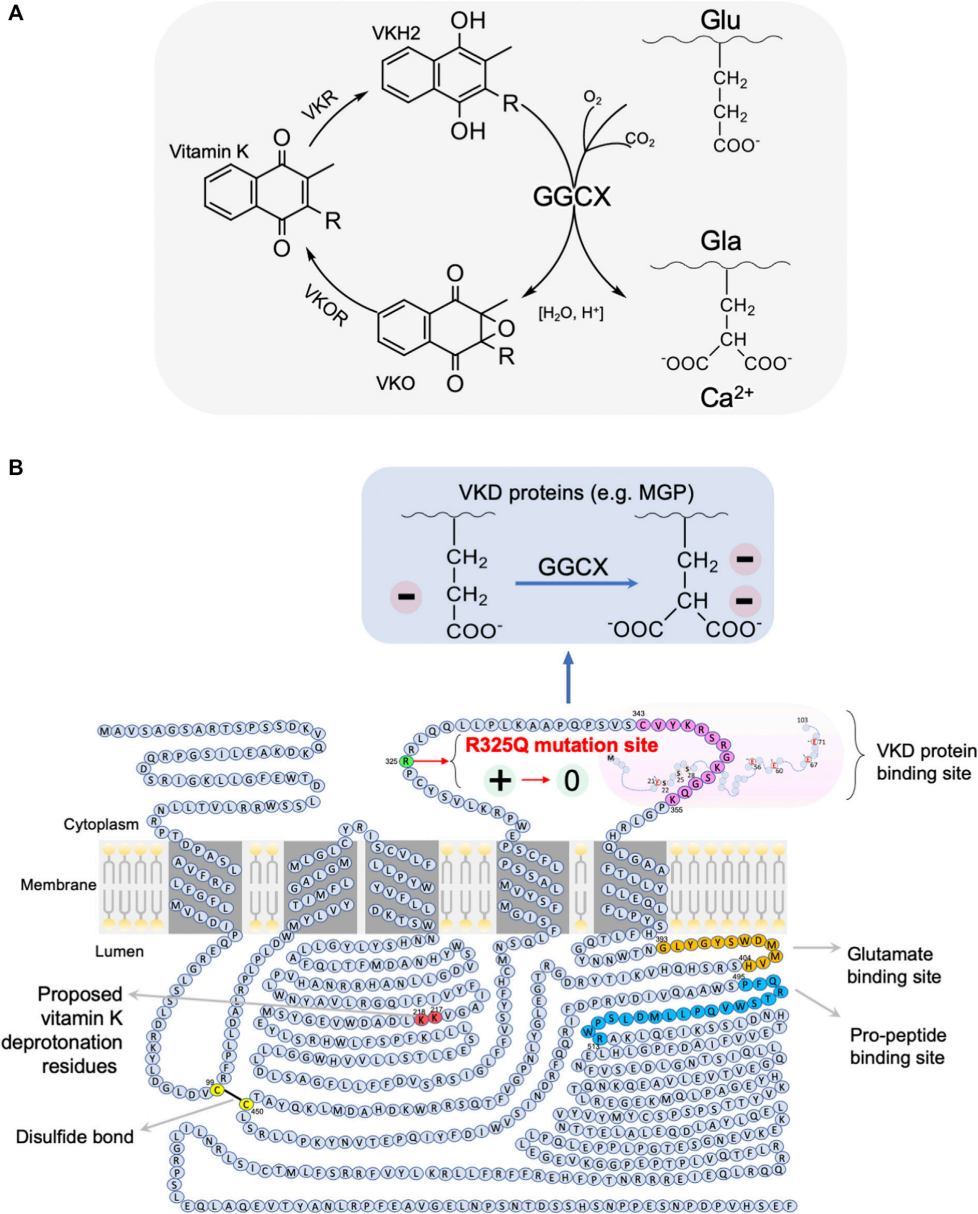
A mechanistic model for the association of the GGCX arginine^325^ to glutamine^325^ (R325Q) SNP with asthenozoospermia infertility risk in men. **(A)** Vitamin K cycle: during vitamin K dependent (VKD) carboxylation, the carboxylase GGCX mediates the pH-dependent conversion of glutamate (Glu) residues to Ca^2+^-binding gamma-carboxyglutamate (Gla) residues of the vitamin K-dependent substrates (e.g., MGP) or their pro-peptides, with CO_2_, O_2_ and reduced vitamin K as the co-substrates. The carboxylation reaction also oxidises the reduced vitamin K-H2 to vitamin K epoxide (VKO), which is then reduced back to vitamin K-H2 by vitamin K epoxide reductase (VKOR). The cycle involves redox- and pH-sensitive carboxylation and oxygenation processes (Rishavy and Berkner, 2012). **(B)** GGCX is a membrane protein with a size of ∼87.5 kDa, which has two intracellular loops and two luminal loops of amino acid sequences, in addition to its cytoplasmic N-terminus and a long luminal C-terminus. The binding sites for glutamate and pro-peptide are located on the long C-terminal tail, which can form a disulfide bond with the first luminal loop of amino acids. It has been hypothesized that all five Glu residues of MGP are carboxylated in one round in sequential order by GGCX, presumably due to MGP stabilisation by the singly positively charged arginine^325^ of GGCX (Rishavy and Berkner, 2012). In our proposed model, the rs699664 SNP-mediated change of arginine^325^ to a neutral glutamine^325^ moiety in the *GGCX* leads to the loss of GGCX binding stability to MGP for carboxylation, thus resulting in decreased carboxylation of VKD proteins, and thereby to increased calcium mineralization and Ca^2+^-mediated proliferation of stress granules, and eventually in a disordered epididymal luminal microenvironment, causing impaired sperm maturation and male infertility. Graphic illustration is adapted from (Tie et al., 2016) with modifications. For more details, see the main text.

As illustrated in Figure 5A, GGCX requires the reduced vitamin KH2 (VKH2) as an obligatory cofactor and its carboxylating activity. Vitamin K generally undergoes a pathway called the vitamin K cycle, in which vitamin K is reduced to VKH2 by vitamin K epoxide reductase (VKOR or VKORC1); VKH2 is then re-oxidized during γ-glutamyl carboxylation by GGCX to vitamin K epoxide (VKO), which is subsequently converted to VKH2 by VKORC1 to complete the VK cycle (Stafford, 2005; Rishavy and Berkner, 2012; Tie and Stafford, 2016).

GGCX is a membrane protein with a size of approximately 87.5 kDa and has five transmembrane domains, with its N-terminus facing the cytoplasmic side and the C-terminus facing the luminal side, in addition to two intracellular loops and two luminal loops, as illustrated in Figure 5B (Tie et al., 2000; Tie et al., 2003; Rishavy and Berkner, 2012; Tie and Stafford, 2016). It is believed that the second intracellular loop contains the binding sequence for the inactive form of MGP (Pudota et al., 2001). On the luminal side, a disulfide bond is formed at position of Cys-99 and Cys-450, which is the essential site for the epoxidation and carboxylation of GGCX (Pudota et al., 2000). This disulfide bond is on the same side as the sequence residues for vitamin K deprotonation (Rishavy et al., 2006) and as the glutamate and pro-peptide binding sites (Mutucumarana et al., 2000; Mutucumarana et al., 2003). On the basis of the nature of this redox-sensitive binding, as well as the epoxidation and deprotonation nature of GGCX, the carboxylation and the other reactions in the vitamin-K cycle are all affected by redox status and acid-base balance (Rishavy and Berkner, 2012). Both of these two physiological factors are known to be essential for sperm maturation and male fertility (Au and Wong, 1980; Carr and Acott, 1984; Breton et al., 1996; Newcombe et al., 2000; Vernet et al., 2004; Pastor-Soler et al., 2005; Aitken and Curry, 2011; Shum et al., 2011; Robaire and Hinton, 2015; Aitken, 2016). It is suspected that the GGCX activity and redox- and pH-sensitive processes in the epididymis reciprocally influence each other.

In our previous study, we identified a single nucleotide polymorphism (SNP), rs699664, in the GGCX gene of infertile men with asthenozoospermia (Ma et al., 2019). This SNP replaces an arginine^325^ with glutamine^325^ (Arg325Gln) in the GGCX protein (Kinoshita et al., 2007; Rieder et al., 2007). The same SNP (Arg325Gln) has been reported to be associated with bone mineral density in aged females, and increased GGCX activity for MGP and bone matrix protein osteocalcin (Kinoshita et al., 2007). The SNP is located in the same second intracellular loop of GGCX, in close proximity to where the binding site of vitamin K-dependent proteins (i.e., MGP) is, as illustrated in Figure 5B. It has been reported that all the five Glu residues of MGP are converted into active Gla in one round of stepwise action of GGCX (Rishavy and Berkner, 2012). This process enables MGP to bind Ca^2+^ ions, Ca^2+^ crystals and bone morphogenetic proteins (Nadeem et al., 2004; Schurgers et al., 2013).

While further investigation is required, we speculate that the positively charged arginine^325^ residue of GGCX is responsible for stabilizing the negatively charged Glu residue of MGP during the stepwise carboxylation process, which is promoted in the acidic conditions of the epididymal lumen. Such charge-charge interaction stability is lost as a result of the SNP, in which the charged arginine^325^ residue is replaced with the neutral glutamine^325^, thereby changing the binding preference of MGP. Accordingly, the charge-charge interaction stability in the wild-type protein ensures sufficient time for complete carboxylation of each residue in the substrates and avoids their incomplete activation (Rishavy and Berkner, 2012). Supporting this interpretation is the finding that the rs699664 SNP in GGCX results in higher carboxylase enzymatic activity in aged females, and was associated with osteoporosis (Kinoshita et al., 2007). We speculate that the rs699664 SNP has similar effects in the epididymis of asthenozoospermic infertile males, i.e., increases early release of incompletely carboxylated substrate, resulting in under-carboxylated MGP, impaired Ca^2+^-binding, disturbed Ca^2+^-homeostasis, and ultimately to impaired sperm maturation and to male infertility.

### A Potential Interplay Between Vitamins in Ca^2+^ Homeostasis in the Epididymis

**FIGURE 6:**
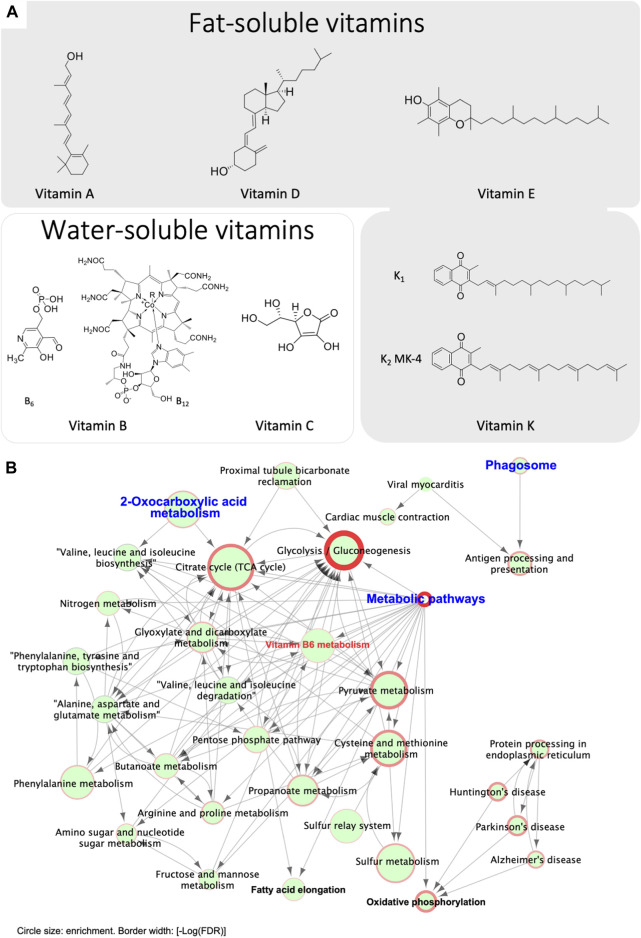
Bioinformatic analysis reveals potential involvement of vitamin B6 pathways in regulating epididymal Ca^2+^ homeostasis and Ca^2+^-dependent MGP-aggregation. **(A)** Chemical structures of the key members of various fat-soluble and water-soluble vitamins. **(B)** Bioinformatic analysis reveals a potential involvement of vitamin B6 in epididymal Ca^2+^ homeostasis. Presented data were originally published in *iScience* (Ma et al., 2019) and reanalyzed. Briefly, proteomic analysis the in-gel digested MGP-positive 32-kDa band was performed with LC-MS/MS analysis (*n* = 3 rats). The MGP-positive 32-kDa band in whole epididymis protein lysates underwent proteomic analysis followed by GO and KEGG-based bioinformatic analysis and identified 301 genes present in all 3 lysates. 2-oxocarboxylic acid metabolism is one of the primary enriched gene clusters, consistent with MGP carboxylation. Other key enriched clusters include phagosome and its downstream pathway of antigen processing and presentation, proximal tubule bicarbonate reclamation, and metabolic pathways. The cluster of metabolic pathways mainly contains sub-clusters for catabolic pathways, including glycolysis and gluconeogenesis, TCA cycle and pyruvate metabolism, in addition to a vitamin-B6 metabolic pathway, suggesting the involvement of vitamin-B6 in the metabolism of Ca^2+^-MGP aggregates. The end products of most of these pathways are substrates for oxidative phosphorylation and fatty acid elongation. These enriched pathways are in consistent with the notion of that chelation activity of carboxylated Ca^2+^-bound MGP contributes to the scavenging of extracellular metabolites, and Ca^2+^ serves as a cofactor in this process. Circle size indicates enrichment degree of each pathway, and border width represents the value of statistical significance of the enriched gene clusters [-Log (False Discovery Rate, FDR)].

An interplay of multiple vitamins may be required for optimal Ca^2+^ homeostasis, including fat- or water-soluble vitamins, as illustrated in Figure 6A. Vitamins B and C are water soluble, whereas other vitamins including vitamins A, D, E and K are all fat-soluble compounds. It has been reported that the systemic action of vitamin D in Ca^2+^ homeostasis can be antagonized by vitamin A, resulting in higher incidents of osteoporosis in man (Johansson and Melhus, 2001). A synergistic interaction between vitamins D and K in bone and cardiovascular health has been proposed, in which vitamin D promotes the production of vitamin K-dependent proteins, including osteocalcin and MGP (Oury et al., 2013; van Ballegooijen et al., 2017). Consistent with this notion, our studies showed that both the vitamin D-related TRPV6-TMEP16A and the vitamin K-dependent GGCX-mediated carboxylation of MGP pathways participate in epididymal Ca^2+^ homeostasis in a spatial complementary manner (Gao da et al., 2016; Ma et al., 2019). To elucidate further the role of vitamin K-dependent Ca^2+^-bound carboxylation-dependent MGP in the epididymal Ca^2+^, we performed a bioinformatic analysis on our published proteome results (Ma et al., 2019), and we found that the vitamin B6-related signaling was also involved in vitamin K-related Ca^2+^-dependent MGP-aggregation, as demonstrated in Figure 6B. These results suggest an interplay of multiple vitamin pathways in regulating epididymal Ca^2+^ homeostasis.

On the basis of the pathway network analysis in Figure 6B, we found that 2-oxocarboxylic acid metabolism as one of the primary enriched gene clusters, consistent with the association of a carboxylation-dependent function of Ca^2+^-bound MGP. In addition, we found that phagosome and antigen processing and presentation as the other key enriched pathways, which were separated from the main primary cluster of metabolic pathways. This may suggest that bound MGP-dependent Ca^2+^-chelation participates in immunological function in the epididymis, which is essential for sperm function (Stammler et al., 2015; Fijak et al., 2018; Voisin et al., 2019). The downstream of metabolic pathways identified in the network analysis are mainly the catabolic pathways, including glycolysis and gluconeogenesis, TCA cycle and pyruvate metabolism. Interestingly, a vitamin B6 metabolic pathway is also one of the identified downstream metabolic pathways, suggesting the involvement of B6 in the metabolism of Ca^2+^-MGP aggregates. The metabolite products of the identified pathways are substrates for oxidative phosphorylation and fatty acid elongation. These results suggested that Ca^2+^-bound MGP aggregates are involved in lipid generation, together with the carbohydrate-related catabolism. In view of the maturing spermatozoa in the epididymal lumen, and the various vitamin-dependent pathways in Ca^2+^-homeostatic regulation, we hypothesized that there is an interplay of different vitamins in the regulation of Ca^2+^ homeostasis in the epididymis, and that the biphasic chelation activity of carboxylated MGP contributes to scavenging of extracellular metabolites, whereas Ca^2+^ serves as the cofactor in this process.

It is known that vitamin K is a key modulator of Ca^2+^ homeostasis in maintaining bone health and for preventing vascular ectopic calcification (Weber, 2001; Wallin et al., 2008; Schurgers et al., 2013), which may also influence male fertility systemically (Karsenty, 2011; Oury et al., 2011; Oury et al., 2013). Since the epididymis is indispensable for sperm maturation, and sperm maturation requires precise regulation Ca^2+^ signaling (Turner, 2002; Weissgerber et al., 2011; Weissgerber et al., 2012; Dacheux and Dacheux, 2014; Robaire and Hinton, 2015), the elucidation of Ca^2+^ homeostasis mechanisms in the epididymis should provide insights into the causes of epididymal-dependent sperm dysfunction and male infertility.

While further investigations are required for a full picture of the interplay between the vitamin signaling pathways, all pathways are connected to Ca^2+^ homeostasis, particularly extracellular Ca^2+^ homeostasis in epididymal fluid. Therefore, low Ca^2+^ is a key factor in regulation of sperm maturation and in male reproductive health. Taken together, the evidence presented here indicates that dysregulated Ca^2+^ homeostasis due to defective expression of Ca^2+^-binding proteins, abnormal Ca^2+^ signaling, or different kinds of vitamin deficiency in the male genital tract may negatively affect male reproductive health.

## Future Perspectives and Conclusion

**FIGURE 7:**
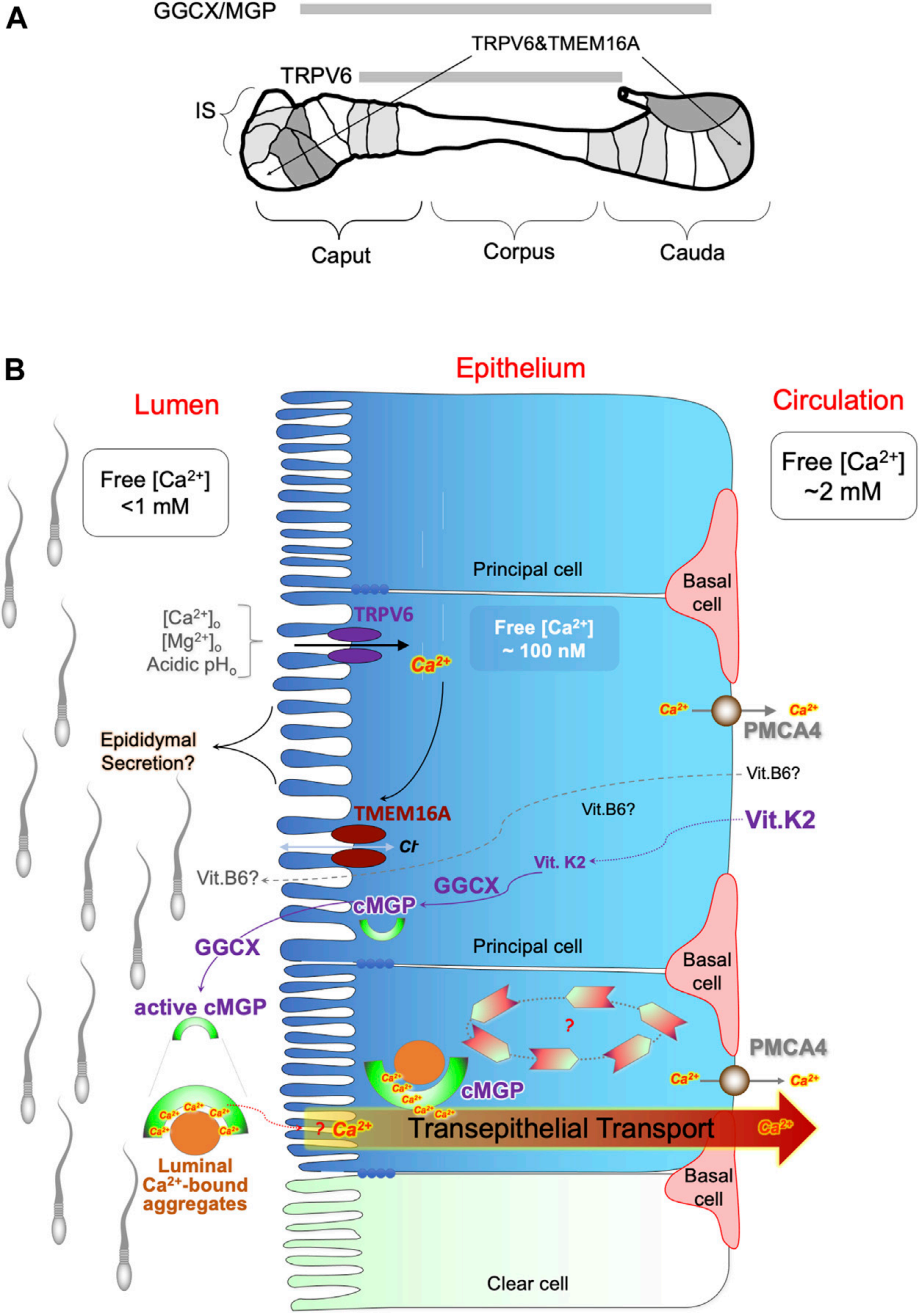
Potential involvement of multiple vitamin pathways in regulation of epididymal Ca^2+^ homeostasis. **(A)** Schematic illustration for the regional expression patterns of the key players regulating Ca^2+^ homeostasis in the rat epididymis, including epithelial Ca^2+^ channel TRPV6, Ca^2+^-dependent chloride channel TMEM16A, γ-carboxylase GGCX and its substrate MGP. IS: initial segment; caput, corpus and cauda indicate head, body and tail region of the epididymis. Spermatozoa mainly mature in the IS and caput epididymidis regions and are stored in the whereas cauda epididymidal region in a dormant stage. **(B)** Schematic diagram of the hypothetical model of Ca^2+^ homeostatic regulation in the epididymal luminal microenvironment during sperm maturation. It involves vitamin D-associated electrical coupling of TRPV6 and TMEM16A channels and vitamin K2-dependent GGCX carboxylation of MGP for luminal Ca^2+^ modulation and protein aggregation. The TRPV6-TMEM16A electrical coupler is involved in fluid transport in a manner of sensitive to extracellular Ca^2+^ and pH. The GGCX-dependent MGP carboxylation plays a role in a spatially complementary manner in the epididymis to promote Ca^2+^-facilitated protein aggregation, thereby maintaining Ca^2+^-homeostasis and pathological calcifications. The potential role of vitamin-B6 in regulating the epididymal luminal microenvironment is also depicted in the proposed model. Ca^2+^-bound MGP-containing protein aggregates in the epididymal lumen act as scavengers in which Ca^2+^ is an essential cofactor for the chelation of Ca^2+^-precipitable metabolites in the extracellular matrix, such as epididymal secretory proteins and shedded remnants from spermatozoa.

Accumulating evidence has indicated the important role of epididymal function in male fertility outcomes, and this function depends to a large degree on the congenial luminal microenvironment established by the epithelial cells lining within the excurrent duct. Important aspects of the luminal microenvironment include Ca^2+^homeostasis of acidity, both of which are strictly regulated by a network of cell-cell interactions and signaling pathways, as depicted in Figure 7. We have provided evidence for the potential role of luminal Ca^2+^ in functioning as a cofactor for the GGCX-dependent carboxylation of MGP, which in turn acts as a scavenger for extracellular metabolites. In addition, we discussed potential interactions of vitamins in these pathways, which are essential for sperm maturation and male reproductive health. This raises the possibility that these multi-vitamin-dependent Ca^2+^ homeostatic-dependent pathways could be leveraged for novel interventions aimed at treating and preventing sperm dysfunctions and male reproductive defecits.
