# Corrigendum: Calcium homeostasis in the epididymal microenvironment: *Is extracellular* c*alcium a cofactor for matrix gla protein-dependent scavenging regulated by vitamins*

**DOI:** 10.3389/fcell.2022.994291

**Published:** 2022-08-24

**Authors:** Winnie Shum, Bao Li Zhang, Albert Shang Cao, Xin Zhou, Su Meng Shi, Ze Yang Zhang, Lou Yi Gu, Shuo Shi

**Affiliations:** ^1^ School of Life Science and Technology, ShanghaiTech University, Shanghai, China; ^2^ Center for Excellence in Molecular Cell Science, Shanghai Institute of Biochemistry and Cell Biology, Chinese Academy of Sciences, Shanghai, China; ^3^ NHC Key Lab of Reproduction Regulation, Shanghai Institute for Biomedical and Pharmaceutical Technologies, Reproduction and Development Institution, Fudan University, Shanghai, China; ^4^ Shanghai Institute for Advanced Immunochemical Studies, ShanghaiTech University, Shanghai, China

**Keywords:** sperm maturation, epididymis, calcium homeostasis, luminal microenvironment, GGCX, matrix gla protein (MGP), TRPV6, TMEM16A

In the published article, there was an error in Table 1 as published. The footnote does not correspond to the superscript in the table. The corrected Table 1 and its caption appear below.

**TABLE 1:** Concentrations of inorganic elements (mM) and pH in blood plasma and intraluminal fluids from the excurrent duct of rats.

	Blood	Seminiferous tubule (SNT)	Rete testis	Efferent duct	Initial segment	Caput	Corpus	Cauda	Vas deferens
Na^+^	138.65∼147.2^a^ ^,^ ^b^ ^,^ ^c^	109.5∼135.44^a^ ^,^ ^b^	130.8∼141.84^b^ ^,^ ^c^ ^,^ ^d^	144.2^c^	136.8^c^	101.8∼112.1^a^ ^,^ ^b^ ^,^ ^d^	57.9∼93.8^a^ ^,^ ^b^	20.6∼37.17^a^ ^,^ ^b^ ^,^ ^d^	23.3^a^
K^+^	4.9∼5.83^b^ ^,^ ^c^	39.77∼46.2^a^ ^,^ ^b^	12.4∼16.1^b^ ^,^ ^c^ ^,^ ^d^	5.7^c^	11.6^c^	16.0∼27.6^a^ ^,^ ^b^ ^,^ ^d^	37.3∼38.3^a^ ^,^ ^b^	39.98∼55.1^a^ ^,^ ^b^ ^,^ ^d^	51.9^a^
Ca^2+^	0.52∼2.4^b^ ^,^ ^c^	0.44^b^	0.81∼0.9^b^ ^,^ ^c^	2.2^c^	1.3^c^	0.85^b^	0.51^b^	0.25^b^	?
Mg^2+^	0.37∼3.3^b^ ^,^ ^c^	1.19^b^	0.39∼1.5^b^ ^,^ ^c^	2.7^c^	1.7^c^	1.97^b^	2.61^b^	0.9^b^	?
Cl^−^	98.0∼122.14^a^ ^,^ ^b^ ^,^ ^c^	118.0∼143.37^a^ ^,^ ^b^	129.7∼135.76^b^ ^,^ ^c^	112.8^c^	116.7^c^	24.25∼31.0^a^ ^,^ ^b^	24.4∼39.09^a^ ^,^ ^b^	23.6∼27.04^a^ ^,^ ^b^	19.3^a^
Total P^e^	2.25∼3.5^b^ ^,^ ^c^	9.22^b^	1.2∼1.72^b^ ^,^ ^c^	3.2^c^	4.5^c^	59.22∼82.5^b^ ^,^ ^f^	80.8∼93.76^b^ ^,^ ^f^	79.4∼88.7^b^ ^,^ ^f^	73.3^f^
HCO_3_^−^	23.0∼30.1^a^ ^,^ ^g^ ^,^ ^h^	10.6∼19^a^ ^,^ ^g^	22.9^h^	45.2^h^	8.7∼20.4^g^ ^,^ ^h^	2.7∼4.8^a^ ^,^ ^g^	?	6.7^a^	6.7^a^
pH	7.39∼7.5^a^ ^,^ ^g^ ^,^ ^h^ ^,^ ^i^	6.93∼7.31^a^ ^,^ ^g^ ^,^ ^i^ ^,^ ^j^	7.34^h^	7.66^h^	6.79∼7.26^g^ ^,^ ^h^ ^,^ ^j^	6.48∼6.64^a^ ^,^ ^g^ ^,^ ^i^ ^,^ ^j^	7.10∼7.18^g^ ^,^ ^i^	6.85∼6.88^a^ ^,^ ^g^ ^,^ ^i^	6.85^a^
Osmolarity^k^	299.4∼311^a^ ^,^ ^c^	338^a^	306.6^c^	303.1^c^	300.5^c^	315^a^	340^a^	329^a^	339^a^

a Data included from **Levine and Marsh (1971)**.

b Data included from **Jenkins et al. (1980)**.

c Data included from **Clulow et al. (1994)**.

d Data included from **Turner (1984)**.

f Data included from **Hinton and Setchell (1980)**.

g Data included from **Caflisch (1992)**.

h Data included from **Newcombe et al. (2000)**.

i Data included from **Caflisch and DuBose (1990)**.

j Data included from **Levine and Kelly (1978)**.

e Total P represents measuremetns including inorganic phosphorus, glycerophosphocholine and phosphocholine.

k Osmolarity unit: mOsm/kg.

In the published article, there was an error in the **Funding** statement. The grant support from “Science and Technology Commission of Shanghai Municipality (STCSM19140903400)” was not included in the original statement, which is:

“This work is supported by NNSFC grants (31871166; 82071704) to WWS and startup of ShanghaiTech University.”

The correct **Funding** statement appears below.

“This work is supported by NNSFC grants (31871166; 82071704), Science and Technology Commission of Shanghai Municipality grant (19140903400), and the startup of ShanghaiTech University to WWS.”

The authors apologize for this error and state that this does not change the scientific conclusions of the article in any way. The original article has been updated.

